# Prediction of Early Distant Recurrence in Upfront Resectable Pancreatic Adenocarcinoma: A Multidisciplinary, Machine Learning-Based Approach

**DOI:** 10.3390/cancers13194938

**Published:** 2021-09-30

**Authors:** Diego Palumbo, Martina Mori, Francesco Prato, Stefano Crippa, Giulio Belfiori, Michele Reni, Junaid Mushtaq, Francesca Aleotti, Giorgia Guazzarotti, Roberta Cao, Stephanie Steidler, Domenico Tamburrino, Emiliano Spezi, Antonella Del Vecchio, Stefano Cascinu, Massimo Falconi, Claudio Fiorino, Francesco De Cobelli

**Affiliations:** 1 Department of Radiology, IRCCS San Raffaele Scientific Institute, 20132 Milan, Italy; palumbo.diego@hsr.it (D.P.); mushtaq.junaid@hsr.it (J.M.); guazzarotti.giorgia@hsr.it (G.G.); steidler.stephanie@hsr.it (S.S.); decobelli.francesco@hsr.it (F.D.C.); 2 School of Medicine, Vita-Salute San Raffaele University, 20132 Milan, Italy; f.prato1@studenti.unisr.it (F.P.); crippa.stefano@hsr.it (S.C.); belfiori.giulio@hsr.it (G.B.); reni.michele@hsr.it (M.R.); aleotti.francesca@hsr.it (F.A.); cao.roberta@hsr.it (R.C.); cascinu.stefano@hsr.it (S.C.); falconi.massimo@hsr.it (M.F.); 3 Department of Medical Physics, IRCCS San Raffaele Scientific Institute, 20132 Milan, Italy; mori.martina@hsr.it (M.M.); delvecchio.antonella@hsr.it (A.D.V.); 4 Pancreatic Surgery Unit, Pancreas Translational and Clinical Research Center, IRCCS San Raffaele Scientific Institute, 20132 Milan, Italy; tamburrino.domenico@hsr.it; 5 Department of Oncology, IRCCS San Raffaele Scientific Institute, 20132 Milan, Italy; 6 School of Engineering, Cardiff University, Cardiff CF24 3AA, UK; espezi@cardiff.ac.uk

**Keywords:** pancreatic adenocarcinoma, X-ray, computed tomography, machine learning, radiomics, prognosis

## Abstract

**Simple Summary:**

If pancreatic adenocarcinoma is assessed to be technically resectable, curative surgery is still suggested as the primary treatment option; however, the recurrence rate can be very high even in this selected population. The aim of our retrospective study was to develop a preoperative model to accurately stratify upfront resectable patients according to the risk of early distant disease relapse after surgery (<12 months from index procedure). Through a machine learning-based approach, we identified one biochemical marker (serum level of CA19.9), one radiological finding (necrosis) and one radiomic feature (SurfAreaToVolumeRatio), all significantly associated with the early resurge of distant recurrence. A model composed of these three variables only allowed identification of those patients at high risk for early distant disease relapse (50% chance of developing metastases within 12 months after surgery), who would benefit from neoadjuvant chemotherapy instead of upfront surgery.

**Abstract:**

Despite careful selection, the recurrence rate after upfront surgery for pancreatic adenocarcinoma can be very high. We aimed to construct and validate a model for the prediction of early distant recurrence (<12 months from index surgery) after upfront pancreaticoduodenectomy. After exclusions, 147 patients were retrospectively enrolled. Preoperative clinical and radiological (CT-based) data were systematically evaluated; moreover, 182 radiomics features (RFs) were extracted. Most significant RFs were selected using minimum redundancy, robustness against delineation uncertainty and an original machine learning bootstrap-based method. Patients were split into training (*n* = 94) and validation cohort (*n* = 53). Multivariable *Cox* regression analysis was first applied on the training cohort; the resulting prognostic index was then tested in the validation cohort. Clinical (serum level of CA19.9), radiological (necrosis), and radiomic (SurfAreaToVolumeRatio) features were significantly associated with the early resurge of distant recurrence. The model combining these three variables performed well in the training cohort (*p* = 0.0015, HR = 3.58, 95%CI = 1.98–6.71) and was then confirmed in the validation cohort (*p* = 0.0178, HR = 5.06, 95%CI = 1.75–14.58). The comparison of survival curves between low and high-risk patients showed a *p*-value <0.0001. Our model may help to better define resectability status, thus providing an actual aid for pancreatic adenocarcinoma patients’ management (upfront surgery vs. neoadjuvant chemotherapy). Independent validations are warranted.

## 1. Introduction

The definition of resectable pancreatic adenocarcinoma is a highly debated issue. The different descriptions proposed over the years are mainly based on the extent of vascular involvement by the tumour [1], which is thought to be the most important factor possibly undermining technical feasibility of resection. According to the 2019 NCCN (National Comprehensive Cancer Network) guidelines [2], resectability status should be determined by a multidisciplinary team that discusses findings on contrast enhanced CT scan and determines if the tumour is (i) resectable, (ii) borderline resectable, (iii) locally advanced/unresectable and (iv) metastatic pancreatic adenocarcinoma. Obviously, different resectability status reflects different scheduled approach and prognosis [2]. However, despite careful selection, approximately 40% of patients undergoing upfront surgery are found to experience distant disease recurrence within 12 months from the index procedure [3], resulting in poor prognosis [3,4].

Overall, these data suggest that upfront surgery is not the best treatment approach for the vast majority of those patients currently being claimed as primary resectable, which could instead benefit from neoadjuvant chemotherapy [5,6]. There is indeed an urgent, unmet need to expand the concept of what is a resectable tumour; along with anatomical definition criteria, some other clinical, pathological and biological features may help in identifying patients who would not benefit from upfront surgery, even when a radiological local disease is present. With regard to this last point, Petrelli and colleagues [3] distinguish between technical and biological resectability, the latter referring to tumours that, despite being technically amenable to surgery, have an unfavourable biology possibly resulting in early relapse and poor survival. Few analyses have been published on this issue, but poor differentiation, high CA19.9 values and long standing symptoms seem to be informative for identifying patients who are likely to have a poor outcome after primary surgery [3,6,7,8,9,10]. However, some limitations undermine this approach, the main ones being the fact that a consistent proportion of patients (around 10% [11]) does not express CA 19.9, and that its absolute value can be affected by concurrent jaundice and/or cholangitis, which are very common occurrences in this population. Perinerual and/or micro vascular infiltration, eventual lymphadenopathies and resection margins status have also been recently advocated as strong predictors of disease-free and overall survival in pancreatic adenocarcinoma patients [12]. However, these pathological findings can be assessed consistently only after resection, lowering their impact on any presurgical decision.

A possible solution to these drawbacks could come from radiomics, a quite novel imaging analysis approach consisting in the extraction of a large amount of quantitative data from medical images [13,14], which may provide a non-invasive, deep insight into tumour microenvironment. However, the application of radiomics to clinical practice is still very limited, mainly due to methodological issues [15] (reliance upon diverse imaging parameters, delineation uncertainty [16], intra- and interscanner variability, need for clinical interpretation of any radiomic signature). With regard to this last point, the selection of few, simple (that means, easily to be interpreted) features is a relevant approach [17,18] when compared to more complex radiomic signatures relying on several features of doubtful clinical significance, frequently weakening validation for clinical use [19,20]. As an example of interpretable features in the setting of pancreatic adenocarcinoma, Choi and colleagues, for instance, reported a correlation between non-complex shape features like irregular margins and DPC4 expression [21].

Given these assumptions, our aim was to apply a robust radiomic approach to derive an usable and interpretable index to identify those patients deemed to be upfront resectable but at high risk for early relapse after surgery, who could instead benefit from neoadjuvant chemotherapy.

## 2. Methods

### 2.1. Patients’ Cohort

This is a single-center retrospective study conducted at San Raffaele Scientific Institute (Milan, Italy); data was collected within the context of an Ethics Committee approved study (28/INT/2015) in patients who had signed an institutional procedure specific informed consent. From a prospectively acquired database, all consecutive patients with pancreatic adenocarcinoma who underwent upfront pancreaticoduodenectomy (PD) between January 2015 and December 2019 were identified (*n* = 652); within this database, patients who were evaluated with at least one multiphase, contrast-enhanced CT scan within 30 days before index surgery (*n* = 156) were enrolled into our study. Patients who died within 90 days after index surgery (*n* = 7) were excluded from further analysis; moreover, two patients had no sufficient follow-up information and were also excluded. The resulting population (*n* = 147) was then randomly split into a training (*n* = 94) and a validation cohort (*n* = 53) according to the second level of the TRIPOD guidelines for the validation of predictive models in oncology [22].

**Figure 1:**
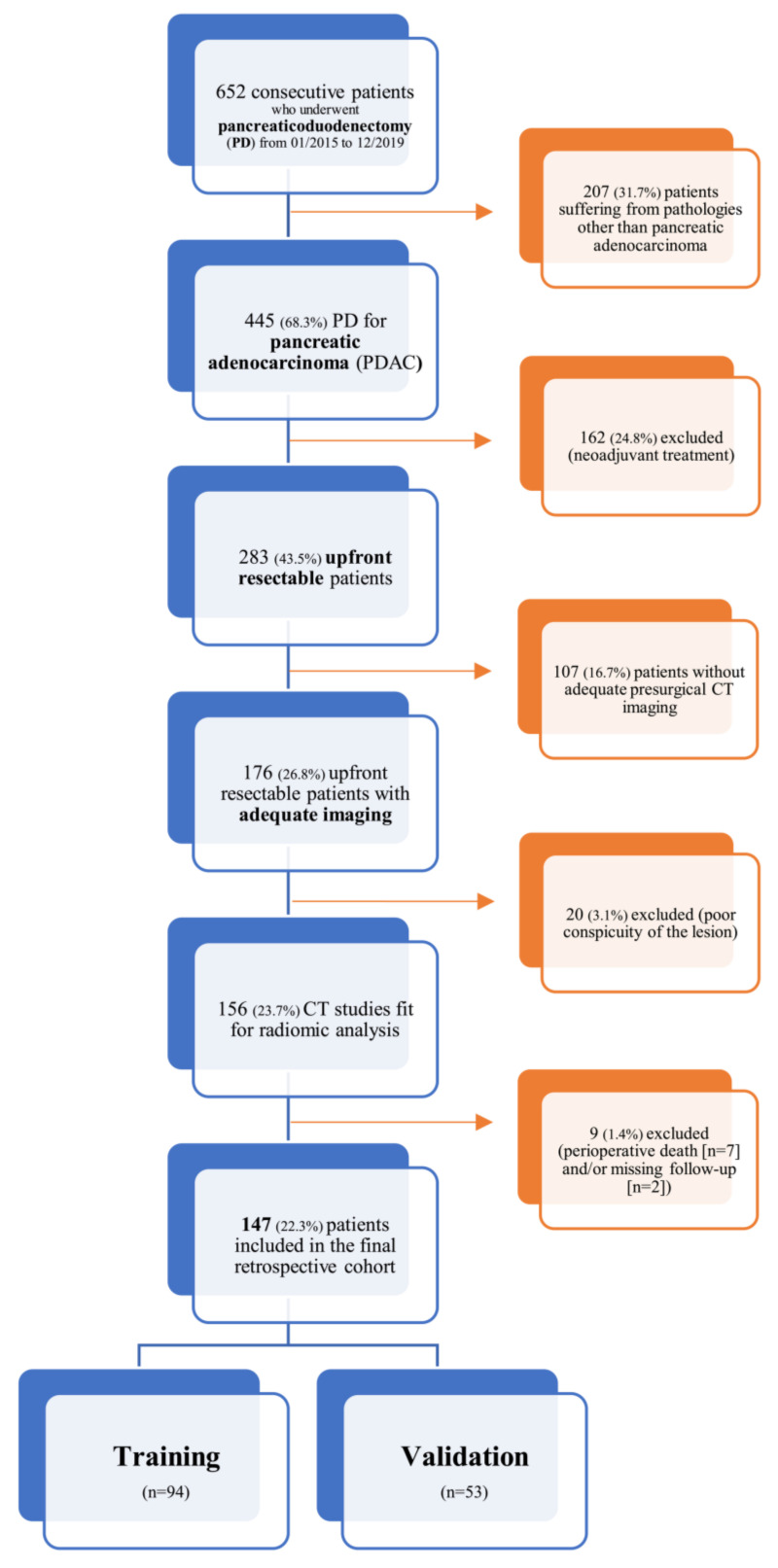
Inclusion and exclusion criteria flowchart.

A detailed flowchart of this study design (comprehensive of inclusion and exclusion criteria) is shown in Figure 1. 

According to the primary endpoint, patients were finally divided into an early distant recurrence (EDR) group (disease free survival < 12 months) and a non-EDR group (disease free survival ≥ 12 months) [3,23,24]; the cut-off was in agreement with the median time to distant relapse observed in our cohort (11 months (IQR: 8–15.7)).

### 2.2. Surgical Technique, Pathology Protocol, Adjuvant Therapy and Follow-Up Data Collection

A multidisciplinary team comprising radiologists, surgeons and oncologists evaluated the included patients and had deemed all of them as upfront resectable according to the 2019 NCCN guidelines [2].

Both pylorus preserving and Whipple PDs were performed by six surgeons with at least 10 years of experience in pancreatic surgery. All patients were treated according to the principles of the Enhanced Recovery after Surgery [25].

Intraoperative frozen examination of the resection margins was performed in all patients, and when positive, the resection was extended, if feasible [26]. After resection, pathologic tumor stage (according to the eighth edition of the American Joint Committee on Cancer staging system [27]), and disease grade were assessed. Perineural invasion was systematically described as present/absent and further classified according to the caliber and number of nerve trunks involved; lymphovascular invasion was also described. The number of metastatic lymph nodes and the ratio of positive to harvested lymph nodes were recorded. Pathological data collected are summarized in Table S1.

Adjuvant treatment was always considered when sufficient recovery within 12 weeks after resection was achieved. All the patients were monitored every three months, until death, via outpatient clinic visits, which included imaging studies and laboratory examinations. Once a follow-up imaging study showed the emergence of any distant lesion, the recurrence was confirmed.

### 2.3. Clinical Variables

Retrospective chart review was used to obtain information on demographics (gender, age, eventual comorbidities), duration of symptoms, laboratory findings and eventual use of adjuvant chemotherapy. The selected clinical variables are summarized in Table S2. Of note, in order to lower possible confounding factors [11], CA 19.9 serum levels were recorded, as a continuous variable, after eventual endoscopic/angiographic palliation.

### 2.4. Radiological Variables and Radiomic Features

In patients who underwent multiple preoperative CT scan, the last examination closest to the date of surgery was used for review.

*CT protocol*—All CT examinations were performed on 64-row multidetector CT scanners (scanner 1: SOMATOM Definition Flash Dual Source CT, Siemens Healthcare; scanner 2: BRILLIANCE, Philips medical system). CT protocol [28] included administration of intravenous non-ionic iodine contrast medium (Iopromide, Ultravist 370 mg iodine/ml (Bayer HealthCare), 120 mL at a rate of 4 mL/s) and consisted of a multiphase acquisition (unenhanced, late arterial, portal venous and late axial scans of the abdomen); axial scans of the thorax were also systematically performed. Scanning parameters were as follows: detector collimation: 64 × 0.62 mm or 128 × 0.6 mm, rotation time: 0.5–0.6, tube voltage: 120 kVp with automated tube current modulation, tube current: 230–300 mAs, slice thickness: 2–3 mm, gap: 1 mm. 

*Conventional Image based parameters*—CT findings were selected for analysis by two radiologists (D.P., F.D.C.) and two senior consultants pancreatic surgeons (S.C., G.B.) on the basis of their clinical experience; variables previously described in the literature were also considered (including those proposed by the Society of Abdominal Radiology and the American Pancreatic Association in their dedicated reporting template [29]). A full list of the selected CT findings is presented in Table S3. Readers with different experiences in abdominal CT imaging were selected for image review: specifically, two residents in their last year of training (J.M., R.C., 4 years experience) and one radiologist (D.P.) with 10 years experience and a subspecialty in abdominal CT imaging. They independently analysed all CT images, blinded to any pathological information. After image review completion, a consensus was established for each selected categorical CT finding; if disagreement existed, the matching results of two readers were chosen for further analysis. 

*Lesion delineation on CT images*—The robustness of CT radiomic features (RF) against interobserver contouring variability was preliminarily assessed on a subgroup of 29 patients by the same three readers. Then, two of these three reviewers contoured all tumour volumes on late arterial phase CT images, where tumour conspicuity was the most. A rigid registration between contrast enhanced and non-contrast enhanced CT images was performed. Contours were transferred from the late arterial to the unenhanced images, and then manually adjusted on the latter to correct minor anatomical discrepancies due to organ motion. Contouring was performed using the MIM Software (v. 6.8.2).

*Radiomic features extraction*—SPAARC Pipeline for Automated Analysis and Radiomics Computing complying with the Image Biomarker Standardization Initiative (IBSI) [15] was used to process images for RF extraction. All images were resampled at 1 mm cubic voxels with a bilinear interpolation. This procedure was implemented to reduce directional bias when voxel sizes were not already isotropic, according to the specific recommendation of IBSI, to allow comparison between image data from different samples, cohorts or batches. This is essential to compare final results because many RF are based on the sum of the entire number of voxels in the lesion. Image rebinning was also necessary, not only to speed up the process of RF extraction, but also to limit noise: we chose 64 bins, as reported in literature [30].

**Figure 2:**
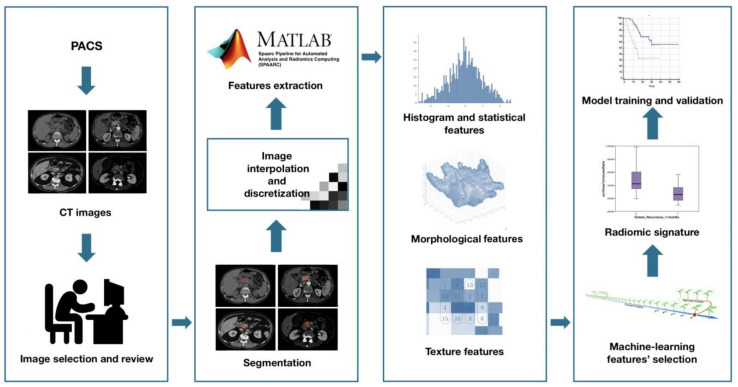
Radiomic features extraction workflow.

Subsequently, adjusted DICOM files were imported to MATLAB using the Computational Environment for Radiological Research. One hundred eighty-two RFs of first and higher order were extracted, belonging to the following families: Morphology, Statistical, Intensity Histogram, Grey Level Co-occurrence Matrix 3D_average (GLCM3D_avg), Grey Level Co-occurrence Matrix 3D_combined (GLCM3D_comb), Grey Level Run Length 3D_average (GLRL3D_avg), Grey Level Run Length 3D_combined (GLRL3D_comb), Grey Level Size Zone Matrix 3D, Neighbour Grey Tone Difference Matrix 3D (NGTDM3D), Grey Level Distance Zone Matrix 3D (GLDZM3D). Figure 2 summarizes the radiomic workflow.

### 2.5. Statistical Analysis

The original population was randomly split into training (*n* = 94) and validation cohorts (*n* = 53) according to the second level of the TRIPOD guidelines for the validation of predictive models in oncology. According to the primary endpoint (EDR evaluated at 12 months), 25 “events” were recorded in the training cohort, making feasible to preferably include a maximum of three variables in the resulting multivariable models [31].

Variables redundancy elimination (Figure S1)—Since the large number of variables (clinical, pure radiologic and radiomic [*n* = 182]) considered, exceeding the number of patients, many variables were expected to be redundant, especially radiomic features owning to the same family. To limit the risk of redundancy, we applied a correlation-based filter: starting from the correlation matrix, a Spearman coefficient (S) threshold equal to 0.70 was arbitrarily fixed to select redundant (S > 0.70) and independent features (S < 0.70). Variables found to be independent were selected; differently, among the redundant variables, the ones with the best p values in Univariate Logistic Regression were selected for further analysis (one for each group of correlated features).

*Inter reader agreement*—The robustness of CT RF against interobserver contouring variability was assessed using intraclass correlation coefficient (ICC), as previously reported [16,32]; ICCs higher than 0.80 were considered to be in high agreement. RF demonstrating an ICC < 0.80 were excluded from further analysis.

Multivariable model development—In order to assess the best combination of the previously selected clinical, radiologic and radiomic variables to predict the primary endpoint, a machine learning bootstrap-based method was used. Briefly, the training set, constituted by the original sample of variables selected, was bootstrapped 1000 times and a backward Univariate Logistic Regression was run for each sample. The variables most significantly associated with the endpoint occurring in each sample were collected and ranked according to their frequency. Accordingly, three models were developed: a strictly radiomic model, a clinicoradiological model, and a combined model considering information from radiomic, conventional radiologic and clinical variables. For each model, the most frequent variables resulting from the bootstrap ranking procedure (taking those variables with *p* value < 0.05 in more than 500 cases on the 1000 bootstrapped samples) were included in a backward multivariable logistic regression for the prediction of EDR. A *p* value < 0.20 and a backward selection were set to retain variables in the model. Finally, a maximum number of variables to be retained equal to three, based on the endpoint events’ number, was fixed.

A prognostic index (P index) was derived for each model according to the following logistic regression formula in the training cohort and then tested in the validation group.

$$Pindex=\frac{1}{\begin{array}{c} 1+\exp-\left(\sum \left(B_{0}*+B_{i}*X_{i}\right)\right) \end{array}}$$

Specifically, for each model the ***B**_i_* coefficients are given applying the logistic regression to the training cohort, whereas ***X**_i_* represents the values of predictors from each patient.

Model performance assessment—To assess the ability of the p index in stratifying patients according to the risk of developing EDR, a cut off value was derived as the best criterion according to the maximum value of the Youden index of the corresponding ROC curve. The P index was then dichotomized as greater or smaller than the cut off value; finally, the separation of the survival curves of the two groups was tested with a Kaplan-Meier test. The P index resulting from the training set was then tested in the validation cohort. The performances of the models were quantified in terms of: area under the ROC curve (AUC), positive and negative predictive values (PPV, NPV), specificity and sensitivity. Analyses were performed using homemade Matlab codes. 

## 3. Results

### 3.1. Patients’ Cohort

Patients’ characteristics are summarized in Table 1. The median overall survival and progression-free survival were 20 (IQR: 15–28) and 15 (IQR: 10–22) months, respectively.

Eighty-five patients out of 147 (57.8%) had a disease recurrence (any sort of (early or late, local or distant)) during the follow-up time period (median follow-up time: 19 months, (IQR: 14–32)). Distant recurrence (*n* = 76, 51.7%) was the most frequent pattern of disease relapse. Considering distant recurrences alone, median time to relapse was 11 months (IQR: 8–15.7), which has been found, in agreement with previous literature [3,23,24], to be a consistent threshold value for distinguishing early and late distant recurrences; accordingly, 39 out of 147 patients (26.5%) included in the final cohort had EDR.

Between training and validation cohorts, no significant differences were found in terms of EDR rate (26.5% vs. 26.3%, *p* = 0.54). No differences were observed when considering both clinical and radiological variables, nor pathological data, except for lymphvascular invasion (93.6% vs. 76.8%, *p* = 0.015). Adjuvant treatment was implemented in 73% (*n* = 69) and 73.2% (*n* = 39) of patients after surgery, respectively per cohort. No significant differences were found in terms of EDR rate between those patients who underwent adjuvant treatment and those who did not (25.7% vs. 30.2%, *p* = 0.197); however, a statistical trend (*p* = 0.068) in favour of adjuvant treatment exists when considering overall disease free survival (16 months (IQR: 10.5–27) vs. 14 months (IQR: 9–22)).

Between EDR and non-EDR groups no statistically significant differences exist in terms of R status (R0 vs. R1) (52.2% vs. 42.2%, *p* = 0.185) and lymphvascular invasion (94.8% vs. 88.5%, *p* = 0.346); EDR group patients had higher lymph node ratio (0.23 ± 0.18 vs. 0.14 ± 0.13, *p* = 0.003).

### 3.2. Variables Selection

**Figure 3:**
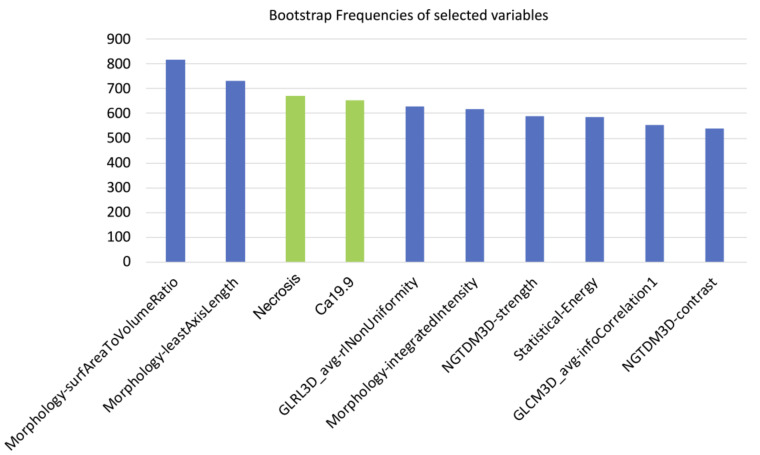
10 most frequent variables identified through a machine-learning bootstrap-ranking procedure (those retaining *p* value less than 0.05 in more than 500 cases on the 1000 bootstrapped samples): eight radiomic features (3 morphologic, 4 texture related and 1 statistical features) and two clinicoradiological variables (radiological necrosis, serum level of CA19.9).

Of the 182 radiomic features extracted, those with ICC values higher than 0.80 (89/182, 48.9%) were considered for further analysis. After further selection to limit the risk of redundancy twenty-three variables were retained and tested. Thereafter, the machine-learning bootstrap-ranking procedure identified the 10 most frequent variables (as explained, those retaining *p* value < 0.05 in more than 500 cases on the 1000 bootstrapped samples): eight radiomic features (3 morphologic, 4 texture related and 1 statistical features) and two clinicoradiological variables. The details of the selected features are shown in Figure 3.

### 3.3. Training and Validation of the Radiomic Model

**Figure 4:**
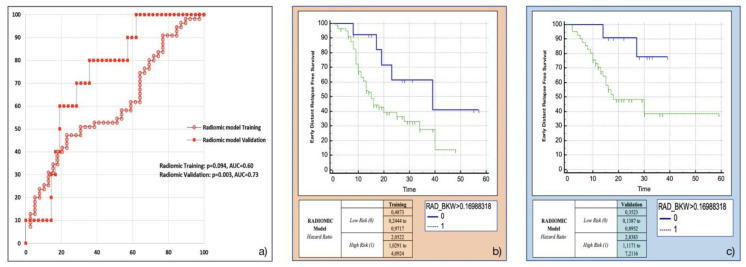
Radiomic model overall performance in terms of (**a**) area under the ROC curve (AUC) for both training (red empty circles) and validation (red filled squares) cohorts, and outcome prediction in terms of Kaplan Meier curve separation between low and high risk patients according to the computed prognostic index in both training (**b**) and validation (**c**) cohorts.

Amongst the eight most frequent RFs resulting from machine-learning bootstrap-ranking procedure, only Surface to Volume ratio was retained in the final model (*p* = 0.0097 (overall fit), AUC = 0.59), with a strong inverse relation (coefficient: −3.82) to the primary endpoint considered. After calculating the corresponding P index, the model was confirmed in the validation cohort (*p* = 0.0244 (overall fit), AUC = 0.73). Further details are provided in Table 2. Corresponding Kaplan-Meier survival curves based on the P index best threshold are shown in Figure 4. With regard to this last point, the radiomic model demonstrated an overall good performance in stratifying the risk of EDR after upfront surgery (training cohort: HR = 2.05, 95% CI = 1.03–4.09; validation cohort: HR = 2.84, 95% CI = 1.12–7.21).

### 3.4. Training and Validation of the Clinicoradiological Model

**Figure 5:**
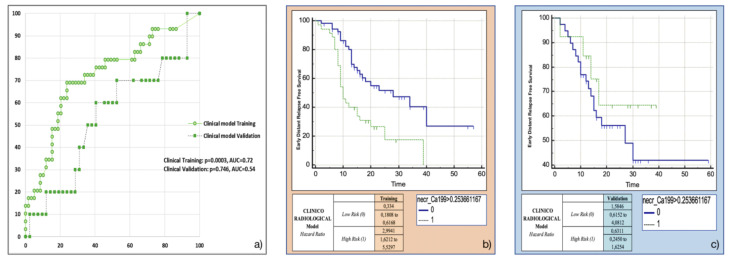
Clinicoradiological model overall performance in terms of (**a**) area under the ROC curve (AUC) for both training (green empty circles) and validation (green filled squares) cohorts, and outcome prediction in terms of Kaplan Meier curve separation between low and high risk patients according to the computed prognostic index in both training (**b**) and validation (**c**) cohorts.

Both the most frequent clinicoradiological variables resulting from machine-learning bootstrap-ranking procedure (presence of tumour necrosis at preoperative CT imaging, and CA 19.9 serum levels) were retained in the final model (*p* = 0.0018 (overall fit), AUC = 0.72). However, after computation of the corresponding P index, the model was not confirmed in the validation cohort (*p* = 0.9529 (overall fit), AUC = 0.54). Further details are provided in Table 2. Corresponding Kaplan-Meier survival curves based on the P index best threshold are shown in Figure 5: the clinicoradiological model failed to predict EDR in the validation set.

### 3.5. Training and Validation of the Combined Model

**Figure 6:**
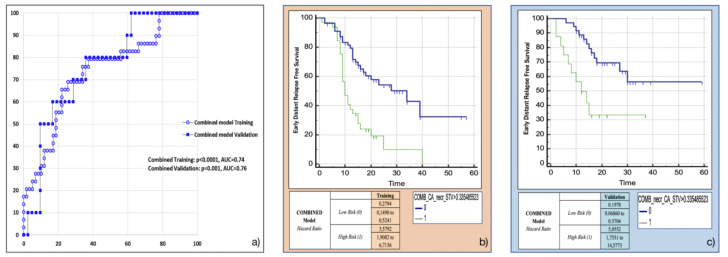
Combined model overall performance in terms of (**a**) area under the ROC curve (AUC) for both training (*blue empty circles*) and validation (*blue filled squares*) cohorts, and outcome prediction in terms of Kaplan Meier curve separation between *low* and *high risk* patients according to the computed prognostic index in both training (**b**) and validation (**c**) cohorts.

The variables retained in this model using the backward multivariable logistic regression were found to be the same as those in the separate models: Surface to Volume ratio, presence of tumour necrosis at preoperative CT imaging and CA 19.9 serum levels. The model developed in the training cohort demonstrated good overall performance (*p* = 0.0015 (overall fit), AUC = 0.75). After calculating the corresponding P index, the model was confirmed in the validation cohort (p = 0.00178 (overall fit), AUC = 0.76). Further details are provided in Table 2. Corresponding Kaplan-Meier survival curves based on the P index best threshold are shown in Figure 6. The combined model demonstrated an excellent performance in stratifying the risk of distant relapse, especially in the first months after upfront surgery (training cohort: HR = 3.58, 95%CI = 1.91–6.71; validation cohort: HR = 5.06, 95%CI = 1.75–14.58): at 12 months after surgery 50% of high risk patients experienced distant relapse of disease vs. 12% of low risk patients (*p* < 0.001).

## 4. Discussion

There is growing literature demonstrating the efficacy of neoadjuvant chemotherapy in patients with resectable pancreatic ductal adenocarcinoma [33]; whether all these patients should receive preoperative chemotherapy remains, though, controversial [5,6,33]. The main issue is that, currently, there is no clinically relevant tool able to accurately stratify patients in terms of early distant relapse (EDR) after upfront surgery. Previously proposed models have limited clinical utility mainly because they consist of pathologic data obtained after surgery and therefore are not applicable in a preoperative setting [6,9,34,35,36,37,38]; another major limitation is the poor, inhomogeneous selection of the study cohorts [6,37,39,40]. In the present study we sought to develop a preoperative model to help identify patients with increased risk of EDR after upfront surgery for pancreatic head adenocarcinoma. To facilitate its use in a clinical setting, only three variables were retained in the final, internally validated combined model: one radiomic feature (Surface to Volume ratio), one conventional radiological variable (presence of tumour necrosis at preoperative CT imaging), and one clinical variable (CA 19.9 serum levels). According to these three variables, a prognostic index can easily be derived for each patient, being a surrogate for the risk of developing EDR after primary surgery. Of note, the combined model outperformed the separate ones (radiomic and clinicoradiological) in terms of (i) overall performance, (ii) robustness and reproducibility, and, above all, (iii) outcome prediction.

Literature has widely described the importance of both radiological tumour necrosis and CA 19.9 serum levels in outlying the biological behaviour of pancreatic adenocarcinomas regardless of anatomical resectability. Kudo and colleagues [35], for instance, identified a worthwhile relation between radiological tumour necrosis and pathological lymph node metastasis and lymphvascular invasion, strongly affecting overall prognosis. On the other hand, CA 19.9 serum levels have been reported to well correlate with disease burden, even besides what imaging can show [3,10,11,41,42,43]. Our results corroborate this evidence. In our cohort, CA 19.9 serum levels have been found to be the most informative clinical predictor of EDR after primary surgery (35 U/mL (non-EDR group) vs. 106 U/mL (EDR group), *p* < 0.001).

On the contrary, the biological significance of the radiomic feature finally retained in our model, Surface to Volume ratio, has not been investigated. It belongs to the morphological family of the radiomic features, summing up the relationship between the surface area of a given object and its volume. Our data highlighted a strong inverse relation between this neoplastic feature and the occurrence of EDR after upfront surgery: in short, adenocarcinomas with low Surface to Volume ratio values were more prone to early relapse after primary surgery. With regard to this last point, one may argue that the assumption that a round shaped tumour with smooth contours (the geometrical object lowering at most Surface to Volume ratio is a sphere) should have a worse prognosis when compared to an ill-defined one is, at least, counterintuitive. In this respect, three reflections have to be done.

Limkin and colleagues [44] demonstrated that the major determinant of Surface to Volume ratio is volume, and therefore it should not be considered as an immediate surrogate for tumour complexity, but rather as a precise tool for dimensional assessment. However, in our cohort, Surface to Volume ratio has been proven to be an extraordinary predictor of EDR, exceeding the other dimension-related variables considered. It follows that Surface to Volume ratio accounts for more information than raw dimensional data do.

According to Bribiesca [45], for similar values of volume, Surface to Volume ratio could be considered an indirect expression of geometrical compactness. Based on our own data, a compact pancreatic tumour (low Surface to Volume ratio) has to be considered at high risk of EDR. Mori and colleagues [18], while developing a PET (positron emission tomography)-based radiomic signature to predict distant relapse free survival in patients with locally advanced pancreatic adenocarcinoma, identified a radiomic feature (Centre of Mass shift), which represents the distance between the geometrical and the metabolic centroids of a given object: the smaller the shift, the more homogeneous the uptake of the contoured lesion and, ultimately, the higher its compactness. The authors found that low values of Centre of Mass shift contributed to worse prognosis, which is in line with our results.

From a biological point of view, Surface to Volume ratio could be considered the major determinant of cell size, since a low ratio may undermine the rate of chemical exchange, resulting in cell death [46]. Moving to tumour perspective, a low Surface to Volume ratio implies impaired vascularization, possibly resulting in tumour hypoxia and necrosis. 

Taken together, these observations allow considering Surface to Volume ratio far more than just a mere morphological feature, and give a novel insight into pancreatic tumour biological behaviour.

However, one may question which is the contribution of Surface to Volume ratio to the overall performance of our combined model. The prognostic index computed from the combined model allowed optimal dichotomization of the validation cohort with 50% of high risk patients experiencing distant relapse of disease within 12 months after surgery vs. 12% of low risk patients (*p* < 0.001). Interestingly, this result was obtained by including a single, extremely robust radiomic feature with a strong biological rationale selected throughout a highly reliable methodological approach. Our approach, hence, differs substantially from previously reported, similar studies [14,40,47] relying instead on several radiomic features ultimately impairing the actual deployment of the resulting models in clinics. Straightforwardness, robustness and reproducibility are, on the contrary, key features of the combined model we propose, which is, moreover, entirely presurgical.

Clinically, our data suggest that those upfront resectable patients thought to be at high risk of EDR according to our combined model should be scheduled to neoadjuvant treatment; on the other hand, low risk patients might be considered a highly selected group possibly suitable for primary resection.

The present study has several limitations, the most important being its retrospective nature and the relatively small number of events observed. External validation is also warranted. Furthermore, our model has been thought not for a standalone usage but rather to be embedded in the multidisciplinary assessment of the patient, which remains the absolute cornerstone in the battleground against pancreatic adenocarcinoma.

## 5. Conclusions

In conclusion, despite the abovementioned limitations, we have developed a robust, entirely preoperative tool to predict early distant relapse of disease after upfront surgery. This model redefines resectability status and provides a personalized tool for patients’ management, identifying those upfront resectable patients at high risk of early recurrence who would benefit from neoadjuvant chemotherapy, as well as those at low risk, which may represent a highly selected group potentially suitable for primary resection. Independent validations of the model are warranted to further corroborate its validity.

## Figures and Tables

**Table 1:** Clinical, pathological and radiological patients’ data and outcome information.

		Total (*n* = 147)	Training (*n* = 94)	Validation (*n* = 53)	*p*-Value
**Clinical Variables**	Age at diagnosis (year) *	69.94 (44–88)	70.06 (43–87)	69.73 (43–88)	0.84
	**Sex**				0.13
	Female	61 (41.6%)	35 (37.6%)	27 (50.5%)	
	Male	86 (58.4%)	59 (63.4%)	26 (49.5%)	
	Ca 19.9 (U/mL) *	40 (14–150)	48.5 (13.25–191)	35 (14.5–77.5)	0.21
	Adjuvant Treatment	111 (75%)	69 (73.2%)	42 (78.6%)	0.38
	Adjuvant Chemoterapy	107 (73.0%)	68 (73.0%)	39 (73.2%)	0.45
	Adjuvant Radioteraphy	31 (21%)	21 (22.3%)	10 (19.6%)	0.11
**Pathological Data**	Tumor Size (mm) ^	27.33 (+/− 0.78)	28.36 (+/− 0.97)	25.48 (+/− 1.29)	0.78
	Final R status				0.08
	R1	65 (44.2%)	44 (46.8%)	21 (39.6%)	
	R0	82 (55.8%)	50 (53.2%)	32 (60.4%)	
	Lymph-vascular Invasion	129 (87.8%)	88 (93.6%)	41 (76.8%)	0.015
	Perineural Invasion	130 (87.1%)	86 (91.4%)	44 (82.1%)	0.06
	Peripancreatic Fat Invasion	135 (91%)	92 (97.8%)	43 (80.3%)	0.72
	Grading				0.42
	G1	3 (2.0%)	3 (3.1%)	0 (0%)	
	G2	66 (45.0%)	45 (47.8%)	26 (48.2%)	
	G3	78 (53.0%)	46 (49.1%)	27 (51.8%)	
	TNM				
	**T**				0.75
	T1	34 (22.7%)	20 (20.3%)	14 (26.8%)	
	T2	102 (69.2%)	68 (72.3%)	34 (64.3%)	
	T3	11 (7.7%)	7 (7.4%)	5 (8.9%)	
	**N**				0.065
	N0	23 (16.1%)	15 (15.0%)	9 (17.8%)	
	N1	50 (33.9%)	26 (27.6%)	24 (44.6%)	
	N2	74 (50%)	54 (57.4%)	20 (37.5%)	
	Lymphnode Ratio *	0.136 (0.04–0.25)	0.16 (0.06–0.26)	0.10 (0.03–0.2)	0.11
**Radiological Data**	Dimension (mm) ^	24.5 (+/− 7.2)	25.79 (+/− 6.8)	23.23 (+/− 7.8)	0.07
	Necrosis				0.34
	Present	27 (18.2%)	18 (19.1%)	9 (16.9%)	
	Absent	120 (81.6%)	76 (85.2%)	44 (83.0%)	
	Hypodense on pancreatic phase	116 (74.4%)	77 (81.9%)	39 (73.5%)	0.48
	Hypodense on venous phase	93 (57.1%)	91 (96.8%)	2 (3.5%)	0.50
	Isodense on pancreatic phase	23 (21.8%)	21 (23.4%)	2 (3.5%)	0.58
**Outcome Variables**	Early distant recurrence (EDR)				0.54
	EDR	39 (25.6%)	25 (26.5%)	14 (26.3%)	
	non-EDR	108 (74.4%)	69 (73.5%)	39 (73.7%)	
	Time to recurrence (months) *	15 (10–22)	15 (9–22)	16 (11–26)	0.55
	Overall survival (months) *	20 (15–28)	20 (15–28)	20 (16–28.5)	0.98
	Length of follow-up (months) *	19 (14–27)	19 (14–27)	19.5 (13.25–27.75)	0.94

Unless otherwise indicated, data are numbers of patients and data in parentheses are percentages. *p* values were determined by comparing characteristics between patients of Training and Validation cohort. * Data are medians, data in parentheses are ranges. ^ Data are means with standard deviations.

**Table 2:** Overall performance in both training and validation cohorts of radiomic, clinicoradiological and combined models quantified in terms of area under the ROC curve (AUC), positive and negative predictive values (PPV, NPV), specificity and sensitivity. OR: Odds Ratio, CI: Confidence Interval.

**Radomic** **Model**		Coefficient	*p*-value	OR	95%CI	**Overall Fit Model**		AUC		95%CI		Sensivity		Specifity		PPP		NPP	
	**Surface To Volume Ratio**	−3.82224	0.0183	0.0219	0.0009 to 0.5237	0.0097	0.0244	0.599	0.733	0.493 to 0.699	0.593 to 0.846	90.9	100.0	23.08	26.2	62.5	24.4	64.3	100.0
**Clinico Radiological Model**		Coefficient	*p*-value	OR	95%CI	**Overall Fit Model**		AUC		95%CI		Sensivity		Specifity		PPP		NPP	
	**Ca19.9**	0.001128	0.049	1.0011	1.0000 to 1.0023	0.0018	0.9529	0.72	0.536	0.614 to 0.811	0.392 to 0.675	68.9	20.0	76.27	73.8	58.8	15.4	83.3	79.5
	**Necrosis**	1.10839	0.0633	3.0295	0.9402 to 9.7611														
**Combined** **Model**		Coefficient	*p*-value	OR	95%CI	**Overall Fit Model**		AUC		95%CI		Sensivity		Specifity		PPP		NPP	
	**Ca19.9**	0.000964	0.0946	1.011	0.9998 to 1.0021	0.0015	0.0178	0.736	0.76	0.648 to 0.838	0.618 to 0.865	58.6	60.0	86.44	78.5	68.0	40.0	81.0	89.2
	**Necrosis**	0.86465	0.1594	2.3742	0.7120 to 7.9167														
	**Surface To Volume Ratio**	−2.8972	0.1171	0.0552	0.0015 to 2.0684														
**Training**																			
**Validation**																			

## Data Availability

Data are available upon request to the corresponding author.

## Supplementary Materials

The following are available online at https://www.mdpi.com/article/10.3390/cancers13194938/s1, Table S1. Pathological variables selected for collection. Table S2. Clinical variables selected for collection. Table S3. Radiological variables selected for collection. Figure S1. Variables selection workflow.
