# Supplementary material for: Prediction of Early Distant Recurrence in Upfront Resectable Pancreatic Adenocarcinoma: A Multidisciplinary, Machine Learning-Based Approach
Source: Cancers (Basel). 2021 Sep 30;13(19):4938. doi: 10.3390/cancers13194938 (PMC8508250; doi:10.3390/cancers13194938)
Supplement: Supplementary file 1 [file cancers-13-04938-s001.zip › cancers-1380678-supplementary.pdf]

SUPPLEMENTARY MATERIALS

Prediction Of Early Distant Recurrence In Upfront Resectable Pancreatic Adenocarcinoma: A Multidisciplinary, Machine Learning-Based Approach

Diego Palumbo; Martina Mori; Francesco Prato; Stefano Crippa; Giulio Belfiori; Michele Reni; Junaid Mushtaq; Francesca Aleotti; Giorgia Guazzarotti; Roberta Cao; Stephanie Steidler; Domenico Tamburrino; Emiliano Spezi; Antonella Del Vecchio; Stefano Cascinu; Massimo Falconi; Claudio Fiorino; Francesco De Cobelli

---

Table S1. Pathological variables selected for collection.

**Tumor size**	absolute value (mm)	**PATHOLOGICAL DATA**
**Final R status**	0 - R0; 1 - R1; 2 - R2	
**Lamina (PV/SMA margin)**	0 - Negative; 1 - Positive	
**Lymphvascular Invasion**	0 - No; 1 - Yes	
**Perineural Invasion**	0 - No; 1 - Yes	
**Peripancreatic Fat Invasion**	0 - No; 1 - Yes	
**Grading**	1 - well; 2 - mod; 3 - scarcely; 4 - anaplastic	
**T**	AJCC 8 ed*	
**N**	AJCC 8 ed*	
**LN Harvested**	absolute value	
**LN Positive**	absolute value	
**LNR**	absolute value	

LNR, lymphnode ratio; *LN*, lymphnode, *PV*, portal vein, *SMA*, superior mesenteric artery
**Chun, Y.S., Pawlik, T.M. & Vauthey, JN. 8th Edition of the AJCC Cancer Staging Manual: Pancreas and Hepatobiliary Cancers. Ann Surg Oncol 25, 845–847 (2018).*

Table S2. Clinical variables selected for collection.

**Ca 19.9**	U/mL	**CLINICAL VARIABLES**
**Age at diagnosis**	years	
**Sex**	0 - Male; 1 - Female	
**Adjuvant treatment**	Adjuvant chemotearpy (0 - No; 1 - Yes)<br>Type of treatment<br>Adjuvant radioterapy (0 - No; 1 - Yes)	

Table S3. Radiological variables selected for collection.

**Tumor Density (HU)**	Basal HU (absolute value)	**RADIOLOGICAL VARIABL**
	Arterial HU (absolute value)	
	Venous HU (absolute value)	
	Arterial/basal HU ratio (absolute value)	
	Venous/basal HU ratio (absolute value)	
**General**	Location (Head *vs.* uncinate process)	

	Size (absolute value, mm)
	Necrosis (0 - No; 1 - Yes)
	Peripheral enhancement (0 - No; 1 - Yes)
	Capsulated (0 - No; 1 - Yes)
	Hypodense on arterial phase (0 - No; 1 - Yes)
	Isodense on arterial phase (0 - No; 1 - Yes)
	Hyperdense on arterial phase (0 - No; 1 - Yes)
	Hypodense on venous phase (0 - No; 1 - Yes)
	Isodense on venous phase (0 - No; 1 - Yes)
	Hyperdense on venous phase (0 - No; 1 - Yes)
	Lymphadenopaties (0 - No; 1 - Yes)
	Fat stranding (0 - No; 1 - Yes)
**Vascular invasion***	SMA contact (0 - No; 1 - Yes)
	Celiac Axis Contact (0 - <180°; 1 - >180°)
	CHA Contact (0 - <180°; 1 - >180°)
	Extension to the celiac axis (0 - <180°; 1 - >180°)
	Extension to the bifurcation of right/left hepatic artery (0 - No; 1 - Yes)
	Arterial Variant (0 - No; 1 - Yes)
	MPV Contact (0 - <180°; 1 - >180°)
	SMV Contact (0 - <180°; 1 - >180°)
	Extension to the most proximal veins draining into the SMV (0 - No; 1 - Yes)
	Thrombus within vein (0 - No; 1 - Yes)
	Venous collaterals (0 - No; 1 - Yes)
**Extrapancreatic extension**	Duodenal invasion (0 - No; 1 - Yes)
	Biliary invasion (0 - No; 1 - Yes)
	Retroperitoneal lamina invasion (0 - No; 1 - Yes)
	Portal invasion (0 - No; 1 - Yes)
	Arterial invasion (0 - No; 1 - Yes)
	Invasion of nearby parenchymal organs (0 - No; 1 - Yes)
**Perineural invasion**	1st plexus capitalis (0 - No; 1 - Yes)
	2nd plexus capitalis (0 - No; 1 - Yes)
	Anterior pathway (0 - No; 1 - Yes)
	Root of mesentery pathway (0 - No; 1 - Yes)
	Number of plexi involved

PV, portal vein; *SMA*, superior mesenteric artery; *CHA*, common haepatic artery; *MPV*, mesenteric portal vein; *SMV*, superior mesenteric vein; *CA*, celiac axis

*NCCN. *NCCN Clinical Practice Guidelines in Oncology. Pancreatic Adenocarcinoma. NCCN Guidelines. (2019).*

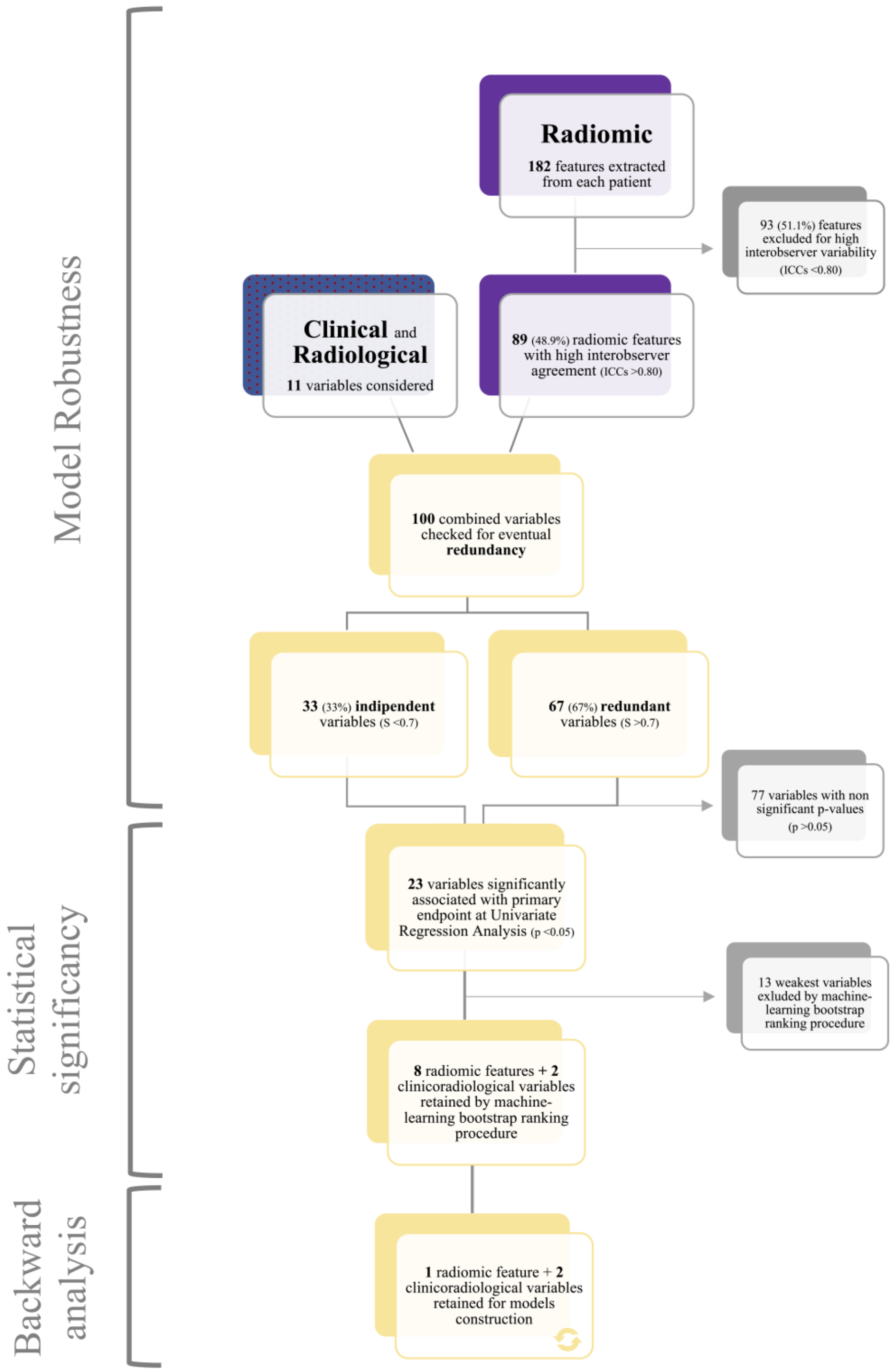

Figure S1. Variables selection workflow.
